# Breakthroughs in Medicinal Chemistry: New Targets and Mechanisms, New Drugs, New Hopes–4

**DOI:** 10.3390/molecules24010130

**Published:** 2018-12-31

**Authors:** Arduino A Mangoni, Catherine Guillou, Jean Jacques Vanden Eynde, Christopher Hulme, Josef Jampilek, Wei Li, Katalin Prokai-Tatrai, Jarkko Rautio, Simona Collina, Tiziano Tuccinardi, Maria Emília Sousa, Jean-Marc Sabatier, Stefania Galdiero, Rafik Karaman, George Kokotos, Giangiacomo Torri, F. Javier Luque, M. Helena Vasconcelos, Dimitra Hadjipavlou-Litina, Carlo Siciliano, Michael Gütschow, Rino Ragno, Paula A. C. Gomes, Luigi A. Agrofoglio, Diego Muñoz-Torrero

**Affiliations:** 1 Discipline of Clinical Pharmacology, College of Medicine and Public Health, Flinders University and Flinders Medical Centre, Bedford Park, SA 5042, Australia; arduino.mangoni@flinders.edu.au; 2 Institut de Chimie des Substances Naturelles, CNRS UPR 2301, Unversité de Paris-Saclay, France; Catherine.guillou@cnrs.fr; 3 Formerly head of the Department of Organic Chemistry (FS), University of Mons-UMONS, 7000 Mons, Belgium; jean-jacques.vandeneynde@ex.umons.ac.be; 4 Department of Pharmacology and Toxicology, and Department of Chemistry and Biochemistry, College of Pharmacy, The University of Arizona, Biological Sciences West Room 351, 1041 East Lowell Street, Tucson, AZ 85721, USA; hulme@pharmacy.arizona.edu; 5 Department of Pharmaceutical Chemistry, Faculty of Pharmacy, Comenius University, Odbojarov 10, SK-832 32 Bratislava, Slovakia; josef.jampilek@gmail.com; 6 Department of Pharmaceutical Sciences, College of Pharmacy, University of Tennessee Health Science Center, 881 Madison Avenue, room 561, Memphis, TN 38163, USA; wli@uthsc.edu; 7 Department of Pharmacology and Neuroscience, University of North Texas Health Science Center, 3500 Camp Bowie Blvd, Fort Worth, TX 76107, USA; katalin.prokai@unthsc.edu; 8 School of Pharmacy, Faculty of Health Sciences, University of Eastern Finland, P.O. Box 1627, FI-70211 Kuopio, Finland; jarkko.rautio@uef.fi; 9 Department of Drug Sciences, Medicinal Chemistry and Pharmaceutical Technology Section, University of Pavia, Viale Taramelli 12, 27100 Pavia, Italy; simona.collina@unipv.it; 10 Department of Pharmacy, University of Pisa, Via Bonanno 6, 56126 Pisa, Italy; tiziano.tuccinardi@unipi.it; 11 Laboratório de Química Orgânica e Farmacêutica, Departamento de Ciências, Químicas, Faculdade de Farmácia, Universidade do Porto, Rua Jorge Viterbo Ferreira 228, 4050-313 Porto, Portugal; esousa@ff.up.pt; 12 Interdisciplinar de Investigação Marinha e Ambiental (CIIMAR/CIMAR), Universidade do Porto, Terminal de Cruzeiros do Porto de Leixões, Avenida General Norton de Matos, S/N 4450-208 Matosinhos, Portugal; 13 Laboratory INSERM UMR 1097, Aix-Marseille University, Parc Scientifique et Technologique de Luminy, 163, Avenue de Luminy, Bâtiment TPR2, Case 939, 13288 Marseille, France; sabatier.jm1@libertysurf.fr; 14 University of Naples Federico II, Department of Pharmacy, Via Mezzocannone 16, 80134 Napoli, Italy; sgaldier@unina.it; 15 Pharmaceutical & Medicinal Chemistry Department, Faculty of Pharmacy, Al-Quds University, POB 20002 Jerusalem, Palestine; dr_karaman@yahoo.com; 16 Department of Sciences, University of Basilicata, Viadell’Ateneo Lucano 10, 85100, Potenza, Italy; 17 Department of Chemistry, National and Kapodistrian University of Athens, Panepistimiopolis, 15771 Athens, Greece; gkokotos@chem.uoa.gr; 18 Carbohydrate Sciences Department, Ronzoni Institute, Milan, Italy; torri@ronzoni.it; 19 Department of Nutrition, Food Sciences and Gastronomy, Faculty of Pharmacy and Food Sciences, Institute of Biomedicine (IBUB) and Institute of Theoretical and Computational Chemistry (IQTC), University of Barcelona, Av. Prat de la Riba 171, E-08921 Santa Coloma de Gramenet, Spain; fjluque@ub.edu; 20 i3S-Instituto de Investigação e Inovação em Saúde, Universidade do Porto, Rua Alfredo Allen 208, 4200-135 Porto, Portugal; hvasconcelos@ipatimup.pt; 21 Cancer Drug Resistance Group-IPATIMUP-Institute of Molecular Pathology and Immunology of the University of Porto, Rua Júlio Amaral de Carvalho, 45, 4200-135 Porto, Portugal; 22 Department of Biological Sciences, FFUP-Faculty of Pharmacy, University of Porto, Rua de Jorge Viterbo Ferreira 228, 4050-313 Porto, Portugal; 23 Department of Pharmaceutical Chemistry, School of Pharmacy, Faculty of Health Sciences, Aristotle University of Thessaloniki, 54124 Thessaloniki, Greece; hadjipav@pharm.auth.gr; 24 Department of Pharmacy, Health and Nutritional Sciences, University of Calabria, I-87036 Arcavacata di Rende, Italy; carlo.siciliano@unical.it; 25 Pharmaceutical Institute, University of Bonn, An der Immenburg 4, 53115 Bonn, Germany; guetschow@uni-bonn.de; 26 Rome Center for Molecular Design, Department of Drug Chemistry and Technology, Sapienza University of Rome, P.le Aldo Moro 5, 00185 Rome, Italy; rino.ragno@uniroma1.it; 27 LAQV-REQUIMTE, Departamento de Química e Bioquímica, Faculdade de Ciências da Universidade do Porto, Rua do Campo Alegre 687, 4169-007 Porto, Portugal; pgomes@fc.up.pt; 28 ICOA UMR CNRS 6005, Universite d’Orleans, Rue de Chartres, 45067 Orleans CEDEX 2, France; luigi.agrofoglio@univ-orleans.fr; 29 Laboratory of Pharmaceutical Chemistry, Faculty of Pharmacy and Food Sciences, and Institute of Biomedicine (IBUB), University of Barcelona, Av. Joan XXIII, 27-31, Barcelona E-08028, Spain

## 1. Introduction

Breakthroughs in Medicinal Chemistry: New Targets and Mechanisms, New Drugs, New Hopes is a series of Editorials, which is published on a biannual basis by the Editorial Board of the Medicinal Chemistry section of the journal *Molecules*. In these Editorials, we highlight in brief reports (of about one hundred words) a number of recently published articles that describe crucial findings, such as the discovery of novel drug targets and mechanisms of action, or novel classes of drugs, which may inspire future medicinal chemistry endeavours devoted to addressing prime unmet medical needs.

## 2. PI3Kδ Inhibition: A New Treatment Paradigm in Asthma and Chronic Obstructive Pulmonary Disease

Highlighted by Arduino A Mangoni

Despite significant advances in diagnosis and treatment, chronic respiratory conditions such as asthma and chronic obstructive pulmonary disease (COPD) remain a major cause of disability and death worldwide [1]. Erra et al report the discovery and the optimization of a series of novel, inhaled, compounds that target the phosphoinositide 3-kinase lipid kinase class I isoform δ (PI3Kδ), primarily involved in the modulation of immune cell function [2,3]. A strategy based on the identification of agents with high potency and selectivity towards PI3Kδ and high plasma clearance led to LAS195319, *N*-[4-(4-{[(1*S*)-1-(5-methyl-4-oxo-3-phenyl-3,4-dihydropyrrolo-[2,1-*f*][1,2,4]triazin-2-yl)ethyl]amino}-7*H*-pyrrolo[2,3-*d*]pyrimidin-5-yl)-1*H*-indol-6-yl]sulfamide. Pharmacokinetic studies in rat showed that LAS195319 exhibited the highest lung exposure, both in solution and in suspension, and lung/plasma partition ratio. In an ovalbumin-induced rat inflammation model, the intratracheal administration of LAS195319 significantly reduced the infiltration of eosinophils and basophils in the bronchoalveolar lavage. Notably the magnitude of these effects was similar to that of fluticasone. Pending further pharmacological and biological characterization, the results of this study suggest that PI3Kδ might represent a promising therapeutic target in asthma and COPD. 

## 3. Design and Synthesis of Tubulin and Histone Deacetylase Inhibitor Based on iso-Combretastatin A-4

Highlighted by Catherine Guillou

In the past decade, the design of multi-target drugs has gained considerable interest due to their benefits in the treatment of complex diseases such as cancer. Their design is considered as a challenge in antitumor drug discovery. In this context, researchers from the CosMIT team (BioCIS-CNRS/UPSud UMR 8076) have designed and synthesized dual molecules. A new series of hybrid molecules based on isocombretastatine A-4 and belinostat was prepared. Two compounds were proven to be potent inhibitors of both tubulin polymerization and HDAC8 activity leading to excellent antiproliferative activity. These compounds have GI_50_ on HCT116 tumor cells in the nM range. They inhibit tubuline polymerization in the μM range and HDAC8 in the nM range [4].

## 4. Expedient On-Resin Synthesis of Peptidic Benzimidazoles

Highlighted by Jean Jacques Vanden Eynde

The lack of new antibiotics and the spread of multi-resistant germs motivate many studies aiming to discover specific targets that are essential for the growth of bacteria. Peptide deformylase is one of those targets. It is a ferrous ion-containing enzyme catalyzing the removal of the *N*-formyl group from *N*-formyl methionine during the protein synthesis. Most inhibitors are pseudopeptide hydroxamic acids, derivatives that can be depicted as peptidomimetics bearing a chelator. Based on that scaffold and because benzimidazoles are antibacterial agents capable of chelating Fe^2+^ ions, benzimidazole conjugates containing amino acids could emerge as alternative inhibitors. Consequently, the elegant solid phase synthesis of such conjugates, as described by Bird et al. [5], appears as an essential tool for the design of novel innovative antibiotics. 

## 5. Development of a Novel Human Parathyroid Hormone Receptor 1 (hPTHR1) Agonist (CH5447240), a Potent and Orally Available Small Molecule for Treatment of Hypoparathyroidism 

Highlighted by Christopher Hulme

PTH is an 84 amino acid polypeptide, secreted by the parathyroid glands, which maintains serum calcium and phosphate levels through agonism of the PTH type 1 receptor (PTHR1) in bone and kidney. The drug teriparatide (Forteo^®^) is a recombinant protein version of PTH consisting of the first (N-terminus) 34 amino acids, the bioactive part of the hormone. As such, it promotes bone growth with utility for osteoporosis. The paper herein details the discovery of a potent, orally bioavailable small molecule PTHR1 agonist, claimed to be the precursor to the clinical candidate PCO371 being evaluated as a treatment for hypoparathyroidism. The translation of 1-34mer peptidic agonistic activity into a small molecule is a significant achievement [6] and if studies are directed towards osteoporosis, the cost of goods should be significantly less than for teriparatide.

## 6. Prospects of Multi-Target Drugs

Highlighted by Josef Jampilek

The new broad-spectrum antibacterial chemotherapeutic 2-{[3-(3,6-dichloro-9*H*-carbazol-9-yl)-2-hydroxypropyl]amino}-2-(hydroxymethyl)propane-1,3-diol (DCAP) specifically disrupts membranes of Gram-positive and Gram-negative bacteria. In addition, it blocks autophagy by preventing autophagolysosome maturation and interrupting the autophagic flux. Autophagy is an important process in adaptive responses to nutrient deprivation and stress, but can be exploited by nutrient-deprived pathogens as well as tumor cells to promote their own survival. The biological effects of DCAP resulted in a significant reduction of uropathogenic *Escherichia coli*, as well as an enhancement of the anticancer activity of the histone acetyltransferase inhibitor vorinostat that increases predisposition to bacterial infections [7]. Thus, DCAP belongs to so-called multi-target agents demonstrating both antibacterial effect and anticancer adjuvant activity. Multi-target drug discovery represents an innovative approach of medicinal chemistry that is based on the concepts of privileged scaffolds, polypharmacology and multifactorial diseases [8]. This strategy seems to be a very useful tool in the design of antineoplastic and anti-infectious agents, as therapeutic agents designed in this way interact with multiple targets and thus prevent resistance acting against various classes of pathogens and simultaneously against tumor cells [9].

## 7. A New Class of Vitamin D3 Analogs That Show High Cell-type Selectivity as Potential Immunomodulatory Agents 

Highlighted by Wei Li

Apart from its classical functions for mineral homeostasis, 1,25-dihydroxyvitamin D3 (1,25D3, the biologically active form of vitamin D3) can produce important immunomodulatory effects that could be useful for treating a variety of diseases such as osteoporosis. However, the use of 1,25D3 for effective immunomodulation is limited mainly due to hypercalcemic toxicities which presumably arise from the activation of the vitamin D receptors (VDR) in intestine cells, instead of selective VDR activations in immune cells. A recent report by Otero and co-workers demonstrated that by proper modifying the sidechain, a new class of vitamin D3 analogs can achieve high cell-type selectivity for kidney cells, bone cells, and monocytes over intestine and skin cells [10]. They further solved the crystal structure of the best compound in complex with the VDR-ligand binding domain and revealed the structural differences in the helix11 region and surface binding, between this compound and the VDR native ligand 1,25D3. These results strongly suggest that it is possible to develop highly cell-type selective VDR activators that can produce strong immunomodulatory effects without inducing the undesired hypercalcemic toxicity. 

## 8. Inhalable JAK1 Inhibitors for Asthma Treatment

Highlighted by Katalin Prokai-Tatrai

According to the World Health Organization, over 200 million people suffer from asthma. There is a certain segment of asthma sufferers that do not respond well to current therapies, calling for alternative therapeutic option. Orally bioavailable Janus kinase 1 (JAK1) inhibitors look promising in animal models of the disease; however, they also cause significant unwanted side effects making them unsuitable for asthma treatment in humans. Recently, a small-molecule JAK1 inhibitor of the pyrazolopyrimidine class called iJak-381 has been reported as a novel approach to treat asthma [11]. iJak-381’s pharmacological action is highly restricted to the lungs after inhalation minimizing therefore systemic side effects. This inhibitor is designed to be rapidly metabolized by the liver in case of accidental systemic exposure, further improving its safety profile. The therapeutic effect of iJak-381 has been tested in various preclinical animal models of asthma convincingly showing that lung-restricted inhibition of JAK1 provides protection against asthma-related inflammation. This suggests that local and selective inhibition of JAK1 has the potential to effectively and safely treat asthma in humans.

## 9. Prodrug Selectively Activated by Gut Enzymes Reduces Abuse

Highlighted by Jarkko Rautio

Stimulant abuse is on the rise and the marketed stimulants are vulnerable to parenteral abuse. This paper describes a novel oral prodrug strategy to resist fencamfamine abuse by preventing intravenous activation of its prodrugs. Prodrugs are mainly activated by pancreatic lipase that is extensively located in the pancreas and gut but only 1% of the enzyme is found in the blood. Especially prodrugs containing long-chain fatty acids (> C_12_) were resistant to intravenous but not oral hydrolysis. The C_17_ chain prodrug demonstrated a near complete intestinal conversion into fencamfamine and stearic acid after oral dose to rats and no intact prodrug was detectable in plasma. In contrast, a substantial amount of intact prodrug was detected in the plasma following the intravenous administration. Finally, the stearic acid prodrug of fencamfamine showed a significant oral but no intravenous increase in locomotion. This study showed, for the first time, that a pancreatic lipase-based mechanism of prodrug activation can address the risk of stimulant abuse. The stearic acid prodrug is being developed for the treatment of apathy in Alzheimer’s disease and binge eating disorder [12].

## 10. Identification of Csf1R as Potential Anti-epileptic Therapeutic Target Applying a Novel Computational Framework 

Highlighted by Simona Collina

The identification and validation of drug target(s) still remains one of the most critical steps in the drug discovery process. Mapping the landscape of a disease in terms of its gene regulatory relationships offers considerable opportunities to accelerate the whole process. To address this problem, the interdisciplinary team coordinated by Prof Michael R. Johnson [13] developed a general computational framework that combines gene regulatory information with causal reasoning (“Causal Reasoning Analytical Framework for Target discovery”—CRAFT). The authors proposed this approach for the identification of cell membrane receptors with a direction-specified influence over disease-related gene expression profiles. As proof of concept, they applied CRAFT to epilepsy and predicted the tyrosine kinase receptor Csf1R as a potential therapeutic target. The effect of Csf1R blockade in attenuating epilepsy seizures in three pre-clinical models of epilepsy was then validated, confirming Csf1R as a novel therapeutic target for counteracting acquired epilepsy. Taken together, these results highlight CRAFT as a systems-level framework for target discovery. The authors suggest that this approach is applicable to disease settings other than epilepsy and therefore CRAFT may pave the way for finding drugs for diseases lacking effective therapy. 

## 11. CDK9 Inhibition as a New Tool for the Reactivation of Epigenetically Silenced Genes in Cancer

Highlighted by Tiziano Tuccinardi

Cyclin-dependent kinase 9 (CDK9) is a member of the positive transcription elongation factor b (P-TEFb) complex which regulates gene transcription elongation by phosphorylating the carboxyl-terminal domain of RNA polymerase II. By using the YB5 cell-based system derived from the human colon cancer cell line as a phenotypic screen, Issa and co-workers identified a potential aminothiazole-based hit compound that was chemically optimized obtaining a highly selective CDK9 inhibitor (MC180295, IC_50_ = 5 nM) characterized by the presence of a bulky norbornyl group linked to the 2,4-diaminothiazole central scaffold [14]. Further studies reported by the authors show that the inhibition of CDK9 determines a reactivation of tumor-suppressor genes and increases sensitivity to immunotherapy in cancer models. Taken together, all these data pave the way for the design and development of new candidate drugs able to selectively inhibit CDK9. 

## 12. Unveiling Lipid Polymorphism as a Mechanism of Membrane-Targeting Antimicrobials

Highlighted by Maria Emília Sousa

Concerning drug targets, lipids have not been as well studied as proteins. However, regarding antibiotics, targeting bacteria cell membrane has been effective for the past 75 years. Could the bacterial membrane still be an attractive target for new antibiotics? Rauter et al. [15] discovered alkyl deoxy glycosides as neutral sugar-based bactericides over Gram-positive bacteria, namely *Bacillus anthracis* and *B. cereus*. In an interdisciplinary approach combining biomolecular simulations on phosphatidylethanolamine (PE)-rich vs. phosphatidylcholine (PC)-rich membranes, differential metabolomics and genomics and biophysical approaches, the authors proposed the unprecedented mode of action of these glycosides to be targeting membrane PE, thereby promoting bacterial membrane disruption through phospholipid lamellar-to-inverted hexagonal phase transition. The authors disclosed this lipid polymorphism being responsible for specific carbohydrate-phospholipid interactions and as a key factor for membrane-targeting antimicrobials. Therefore, the bacterial membrane can be considered an attractive target, based on its essentiality, differential arrangement from mammalian membranes, and its “resistance to resistance”.

## 13. Structure-Activity Relationship Study of a Social Wasp’s Venom Antimicrobial Peptide (polybia-CP) Highlights Some “Key” Functional Motifs Allowing to Design Novel Candidate Antibiotics

Highlighted by Jean-Marc Sabatier

The extensive use of antibiotics has favored the emergence of multidrug-resistant bacteria. Antimicrobial peptides (AMPs) from the venoms of insects and arachnids are promising candidates to potentially treat drug-resistant bacterial infections. Torres and collaborators [16] used a structure-function relationship strategy in the AMP polybia-CP (*Polybia paulista* wasp venom) to define its “key” functional motifs/hotspots. The helical content, net positive charge, hydrophobicity, and hydrophobic moment were highlighted as “key” determinants of its antimicrobial potential. The data were used to generate new AMPs with antibacterial activity in a mouse model. The approach described, referred to as a physicochemical-guided rational design strategy to generate peptide antibiotics, suggests that fine-tuning of such “key” parameters will enable to design nontoxic but potent AMPs with improved broad-spectrum antibacterial properties in vitro and in vivo. 

## 14. Nanofiber Technology: A Bioinspired Challenge for Treatment of Cancer

Highlighted by Stefania Galdiero

The development of drug nanocarriers for cancer is extremely complex. Bellat et al. [17] report on a novel biocompatible, non-immunogenic formulation of self-assembling peptide nanofiber precursors (NFP) characterized by a high design flexibility which allows for different lengths, charge distributions, drugs, imaging agents, chemical moieties and functional domains. Furthermore, the optimized glutathione (GSH)-NFP shows a unique combination of tumor uptake, penetration, infiltration, residing and retention properties. Thus, the therapeutic and safety benefits of the formulation able to release doxorubicin within the acidic environment of the tumor are also reported. An innovative and fascinating approach for further developments in next generation drug nanocarriers for combination therapies to treat cancers and infectious diseases is described. This groundbreaking paper suggests that these combinatorial approaches are entirely feasible.

## 15. Odilorhabdins, Antibacterial Agents that Cause Miscoding by Binding at a New Ribosomal Site

Highlighted by Rafik Karaman

A new class of antibiotics has been published by researchers from the USA and France. This class, odilorhabdins, or ODLs, is unique and promising due to the fact that its source is unconventional and it has a unique way of killing bacteria. Therefore, it is believed that these antibacterials may be very efficient at treating drug-resistant bacterial infections. The ODLs were produced by symbiotic bacteria spotted in soil-dwelling nematode worms colonizing insects for food. The study revealed that ODLs act by targeting the ribosome. However, their mechanism of action is unique because they bind to a place on the ribosome that has never been used by other known antibiotics. The ODLs were found to bind to the ribosome; the antibiotic disrupts the ability of the bacteria to interpret and translate genetic code causing the ribosome to make mistakes when it creates new proteins. This miscoding leads to corruption of the cell and causes the bacterial cell to die. The unique mechanism of ODLs is a very strong indicator that ODLs have the potential to treat infections that are untreated by other antibiotics [18]. 

## 16. A Novel Role of Ceramide Synthase 1 in Fat Metabolism Has Been Defined Using a Selective Inhibitor 

Highlighted by George Kokotos

Ceramides are a family of bioactive lipids, which are involved in lipid signaling. They regulate several physiological functions, including insulin sensitivity, and their increased levels have been implicated in the pathogenesis of insulin resistance. In mammals, the biosynthesis of ceramides is accomplished via the action of six different ceramide synthases (CerS1-6). C18 ceramide, which is synthesized by ceramide synthase 1 (CerS1), has been suggested to promote insulin resistance in humans. Recently, the discovery and characterization of the first potent, isoform-selective CerS inhibitor, which specifically targets CerS1, has been reported [19]. P053, (*S*)-2-amino-4-(4-(3,4-dichlorobenzyloxy)phenyl)-2-methylbutan-1-ol, inhibits CerS1 with nanomolar potency, exhibiting selectivity over the other CerS isoforms, as demonstrated by lipidomic profiling. Using this inhibitor, a role for CerS1 as an endogenous inhibitor of mitochondrial fatty acid oxidation in muscle and regulator of whole-body adiposity has been defined. CerS1 is involved in fat metabolism and its inhibition prevents fat deposition, but not insulin resistance induced by a high-fat diet.

## 17. Heparanase Inhibitor Clinical Trials

Highlighted by Giangiacomo Torri

Heparanase is a mammalian endoglycosidase that is overexpressed in various tumor forms. The overexpression of heparanase by tumor cells is mainly correlated with their ability to form metastases and with the vascularization of the tumor mass which ends with the reduction of survival expectations of cancer patients also after surgical treatment. In animal models it has been observed that non-anticoagulant heparins and their mimetics, such as sulfate oligosaccharides, inhibit the action of heparanase, thereby becoming promising antimetastatic and antitumor drugs in various solid tumor diseases. The inhibitory action of these drug candidates, whose chemical structure has been widely studied, depends on the fact that they block the active site of this hydrolase mimicking the sequence glucuronic acid β1-4 α glucosamine present in the heparan sulfates of the cell membranes. Substances labeled with laboratory codes PI88, PG545, and M402 have passed Phase 1 clinical trials. A recent publication reports the first trial evaluating a heparanase inhibitor in hematologic malignancies. The results of the study on SST0001, a derivative of glycol split heparin having as its druggable target multiple myeloma, show a safety and high tolerability profile [20].

## 18. Binding Kinetics Survey of the Drugged Kinome

Highlighted by F. Javier Luque

Since the introduction of the drug–target residence time model around a decade ago, increasing attention has been paid to the lifetime of drug–target complexes to understand the activity of small compounds [21]. Georgi and coworkers report an interesting application to kinase inhibitors [22]. To this end, the authors performed high-throughput binding kinetics assays to characterize the interaction of 270 compounds (32 FDA-approved drugs and 238 clinical and preclinical candidates and tool compounds) with 40 clinically-validated kinases. The results revealed that on-rates (k_on_) are better correlated with dissociation constants (K_D_) than off-rates (k_off_). However, this trend was gradually inverted with the inhibitory potency, the fraction of slowly dissociating compounds being increased upon transition from early to late stages in drug discovery. In addition, the interplay between target occupancy and the plasma elimination rate is critical for the efficacy profile of drugs, being relevant for achieving kinetic selectivity between primary and potential off-targets. Finally, hydrophobic interactions seem to play an important role in dictating the slow dissociating behavior of compounds. Altogether, this study underscores the impact of binding kinetics in providing clues for the prospective design of novel bioactive compounds.

## 19. Doxycycline, an Inhibitor of Mitochondrial Biogenesis, Effectively Reduces Cancer Stem Cells (CSCs) in Early Breast Cancer Patients: A Clinical Pilot Study

Highlighted by M. Helena Vasconcelos

Targeting cancer stem cells (CSCs) may be an exceptional approach to prevent and treat cancer, avoiding tumor relapse. CSCs may be responsible for tumor initiation, maintenance, metastasis, resistance to therapeutic approaches and recurrence. However, finding specific targets and discovering drugs that specifically target CSCs has proved a hurdle. Remarkably, different mitochondrial functions were reported to promote acquisition and maintenance of a CSC phenotype [23], suggesting that inhibition of mitochondrial function could be the “Achilles heel” of CSCs. This prompted novel drug-discovery and also drug-repurposing studies, since mitochondria evolved from bacteria and some of the currently clinically-used antibiotics target mitochondria as a side effect. The recent publication by Scatena C. et al. [24] shows results from the first clinical pilot study using short-term pre-operative treatment with oral doxycycline (a broad-spectrum antibiotic) to eliminate CSCs in early breast cancer patients. Preliminary results indicate that doxycycline can reduce the expression of CD44 and ALDH1, two putative CSC markers. If confirmed, this could signify a major breakthrough in cancer treatment, by repurposing cheap, over-the-counter antibiotics to be used together with standard treatments, to achieve long-term survival of patients. 

## 20. Target Validation and Identification of Novel Boronate Inhibitors of the *Plasmodium falciparum* Proteasome

Highlighted by Dimitra Hadjipavlou-Litina

Malaria remains a major health problem for millions of people. The threat is increasing due to the climatic changes on the planet. Current antimalarial therapies are highly dependent on artemisinin-based combination therapies. Proteasome inhibitors show potential for the treatment of malaria, exhibiting parasiticidal activity. Bortezomib, a covalent peptide boronate proteasome inhibitor which was used clinically to treat multiple myeloma, has been shown to have activity against *P. falciparum*. Tilley et al. [25] validated the β5 subunit of the proteasome as the target to generate bortezomib-resistant parasites. A boronate peptide library was screened to identify inhibitors of the growth of cultures of *P. falciparum*. It seems that inhibition of β5 activity is needed for potent antiplasmodial activity. Amino acid residues at the P1, P2, and P3 can confer selective binding to the *P. falciparum* proteasome, providing a path to further modification of the peptide boronate scaffold in order to generate more selective inhibitors. The profiling of the substrate reveals *P. falciparum* 20S proteasome active site preferences that will inform attempts to design more selective inhibitors.

## 21. Discovery of Novel and Powerful Antidepressants

Highlighted by Carlo Siciliano

The discovery of antidepressants is arguably the greatest breakthrough in the field of major depression. This disorder is one of the most common, burdensome, and costly psychiatric disorders worldwide, causing an elevated risk of suicide. Unfortunately, depression is compounded by the lack of fast and efficacious pharmacological treatments. Antidepressants inhibit MAO-A, but they are not selective because also MAO-B is inhibited in variable extent. These deficiencies highlight the need for the development of novel medicaments. The discovery of the serotoninergic 5-HT_7_ receptor unlocked new potentialities in the clinical treatment of depression. Synthetic hydantoin derivatives have been designed as potent and selective serotonin 5-HT_7_ receptor agents [26]. 5-Phenyl-3-(2-hydroxy-3-(4-(2-ethoxyphenyl)piperazin-1-yl)propyl-5-methylimidazolidine-2,4-dione was the base structure. All derivatives were easily prepared, characterized, their crystal structures were obtained, and comprehensive investigations performed by computer-aided studies established their structure-ligand interactions, affinities and selectivities. All hydantoin analogues showed potent affinities and selectivities, and their antidepressant-like effects were investigated in vivo by behavioural tests. Aryl substituents on hydantoin and piperazine scaffolds generated powerful antidepressants whose strong benefits on CNS were definitively assessed in silico.

## 22. Rational Tuning of Fluorobenzene Probes for Cysteine-Selective Protein Modification

Highlighted by Michael Gütschow

In this impressive study by Morten Meldal, Frederik Diness and their coworkers at the Center for Evolutionary Chemical Biology, Department of Chemistry, University of Copenhagen, novel probes for site selective cysteine labeling have been introduced [27]. The authors performed a reactivity tuning of fluorobenzenes towards S_N_Ar reactions with Boc-cysteine which relied on the electron withdrawing capacity of the *para* substituent and on the number of fluorine substituents introduced. Optimized probes selectively arylated cysteine residues in proteins under aqueous conditions. Chemoselective inhibition of a cysteine protease, papain, over a serine protease, subtilisin, was also demonstrated. Noteworthy, fluorobenzene probes allowed discrimination among cysteine residues with different physicochemical properties in a model protein, tobacco etch virus protease. The novel probes were successfully applied for activity-based protein profiling and for identifying proteins containing reactive cysteine residues in cellular lysates.

## 23. The PROTAC Approach as a Promising Chemical Weapon in Epigenetic Modulation

Highlighted by Rino Ragno

PROTACs (proteolysis targeting chimeras) are small bi-functional molecules designed to induce the degradation of a target protein through a proteasome-dependent mechanism [28]. In the last decade several PROTACs have been successfully applied demonstrating to be a robust strategy to generate potential novel therapeutics useful also for undruggable targets [29]. In particular, Bassi et al. developed novel potential anti-inflammatory therapeutic agents by generating the first PROTACs directed to the degradation and modulation of P300/CBP-associated factor (PCAF) and general control nonderepressible 5 (GCN5) epigenetic proteins, containing an acetyltransferase domain and a bromodomain [30]. Their synthesized racemic and pure enantiomers of designed PCAF PROTACs through extensive biochemical and biological evaluation were proved to be effective in downregulating PCAF protein levels at over 90% in macrophages and monocyte-derived dendritic cells. Data revealed the approach to be a promising one to extend the tractability of nuclear proteins, such as epigenetic targets, to small molecule modulation able to selectively bind to pre-defined targets. No matter what the physiological mechanism of PROTACs could be, this strategy represents a way to develop effective small molecule focused degraders.

## 24. Synthesis of New Betulinic Acid/Betulin-Derived Dimers and Hybrids with Potent Antimalarial and Antiviral Activities

Highlighted by Paula A. C. Gomes

In tropical and sub-tropical areas of the globe, malaria is a leading cause of morbidity and mortality, especially amongst young children. Prognosis is also very poor for people co-infected with malaria and other pathogens, especially, retroviruses like HIV-1 or herpetic viruses, like the human cytomegalovirus (HMCV) [31,32]. As such, any developments towards promising candidates to fight malarial-viral co-infections are of utmost importance to tackle this tremendous health burden. In this connection, the recent report by Tsogoeva and co-workers [33] is an important first step towards that end. In this work, betulinic acid/betulin based dimer and hybrid compounds carrying ferrocene and/or artesunic acid moieties displayed potent in vitro action against both *P. falciparum* and HCMV. Additive and/or synergistic effects were also observed between the natural or semisynthetic products, such as betulinic acid-/betulin- and artesunic acid-derived compounds. Overall, these are relevant findings towards future development of effective dual-action medicines against co-infections.

## 25. Small Drug TH5487 Inhibits OGG1 and Acts as Potent New Anti-inflammatory 

Highlighted by Luigi A. Agrofoglio

A new small drug candidate, TH5487 [4-(4-bromo-2-oxo-2,3-dihydro-1*H*-benzo[*d*]imidazol-1-yl)-*N*-(4-iodophenyl)piperidine-1-carboxamide], was reported by I. Boldogh, T. Helleday and collaborators to be a selective active-site inhibitor of 8-oxoguanine DNA glycosylase 1 (OGG1) [34]. This new compound suppresses proinflammatory gene expression and inflammation in mice. Because OGG1 binds 8-oxoG and because OGG1-deficient mice are resistant to acute and systemic inflammation, authors hypothesized that OGG1 inhibition may represent a strategy for the prevention and treatment of inflammation. TH5487 inhibits DNA repair by preventing OGG1 from binding to its DNA substrate and does not affect the activity of other DNA glycosylases. This proof of concept will help in designing small molecules that targeting oxidative DNA repair can alleviate inflammatory conditions in vivo.

## 26. A New Prodrug Strategy for Sulphate Containing Drugs Enables the Development of New Oral β-Lactamase Inhibitors

Highlighted by Diego Muñoz-Torrero

Thirty-five years after launch, clavulanic acid remains the only approved oral β-lactamase inhibitor (BLI). Diazabicyclooctanones (DBOs) are a new class of non-β-lactam BLIs but the presence of a sulfate group in their structure precludes oral absorption, making it necessary a much less convenient intravenous administration. Gordon et al. have developed a very elegant strategy for the design of orally available prodrugs of the FDA-approved DBO avibactam [35]. The new prodrug strategy combines the stability of neopentyl groups as sulfate protecting groups [36] with the metabolic lability of an ester group placed at the end of the side chain, which upon in vivo esterase cleavage reveals an appropriately placed nucleophile (carboxylate or alkoxide) that intramolecularly attacks the electrophilic neopentyl methylene group, thereby expelling the active drug. The steric hindrance exerted by the gem-dimethyl group prevents alkylation of external nucleophiles (biomolecules) while favoring the attack of the intramolecular nucleophile by bringing it closer to the displacement site. The novel prodrugs display high oral bioavailability in rat, dog and monkey, and in combination with appropriate oral β-lactam antibiotics restore the antibiotic’s effectiveness.
